# Guided endodontics versus conventional access cavity preparation: an ex vivo comparative study of substance loss

**DOI:** 10.1186/s12903-023-03436-7

**Published:** 2023-10-04

**Authors:** Hauke Hildebrand, Wadim Leontiev, Gabriel Krastl, Roland Weiger, Dorothea Dagassan-Berndt, Sebastian Bürklein, Thomas Connert

**Affiliations:** 1 Department of Periodontology, Endodontology and Cariology, University Center for Dental Medicine, University of Basel, Basel, Switzerland; 2 Department of Conservative Dentistry and Periodontology, University Hospital of Würzburg, Würzburg, Germany; 3 Center for Dental Imaging, University Center for Dental Medicine Basel UZB, University of Basel, Basel, Switzerland; 4 Interdisciplinary Ambulance in the School of Dentistry, Albert-Schweitzer-Campus 1, 48149 Münster, Germany

**Keywords:** Endodontic access cavity, Pulp canal calcification, Surgical planning software, Pulp canal obliteration

## Abstract

**Background:**

To compare the outcomes of conventional access cavity preparation (CONV) versus guided endodontics (GE) for access cavity preparation in anterior teeth with pulp canal calcification (PCC) regarding root canal detection, substance loss, procedural time, and need for additional radiographs.

**Methods:**

Extracted, sound human teeth with PCC (n = 108) were matched in pairs, divided into two groups and used to produce 18 models. An independent endodontist and a general dentist performed access cavity preparation under simulated clinical conditions on nine models each (54 teeth). The endodontist used the conventional technique and the general dentist GE. Time needed to access the root canals and the number of additional radiographs were recorded. Pre- and postoperative cone-beam computed tomography scans were obtained to measure substance loss. Statistical significance was tested by examining the overlap of 95% confidence intervals (CIs) between the groups.

**Results:**

All root canals were successfully accessed by both methods. There were no significant differences in substance loss (CI: CONV 15.9–29.6 mm^3^ vs. GE 17.6-27.5mm^3^) or procedural time (CI: CONV 163.3-248.5 s vs. GE 231.9-326.8 s). However, 31 additional radiographs were required for GE compared to none for CONV.

**Conclusions:**

For access cavity preparation in teeth with PCC, both CONV by a specialist and GE by a general dentist produce good results in terms of substance loss and time requirements.

**Supplementary Information:**

The online version contains supplementary material available at 10.1186/s12903-023-03436-7.

## Background

Teeth with calcified root canals present a significant treatment challenge in endodontics. The reasons for pulp canal calcification (PCC) are manifold [1]. Frequently, PCC develops asymptomatically and is discovered incidentally during routine checkups. Clinically, a discolored tooth is usually seen, initially indicating vital pulp tissue. Treatment of these teeth is not indicated until they show clinical signs of a pulpitis or apical pathology radiologically [2, 3]. This is the case in 7–27% of teeth with PCC after suffering a dental trauma in the course of time [4].

Methods for treating teeth with PCC teeth have improved in recent decades with the introduction of new techniques. Dental microscopes and guided endodontics (GE) offer important assistance to the practitioner [5, 6]. The use of dental microscopes allows endodontists to find even severely calcified root canals by conventional access cavity preparation (CONV) in up to 90% of cases [5]. As an alternative treatment method, GE guides a drill to the orifice of a calcified root canal using a template, based on a three-dimensional (3D) X-ray image and an intraoral scan.

GE gives general dentists the potential to treat complex cases. Several studies investigating the success rates of GE versus conventional freehand methods for access cavity preparation (ACP) in 3D printed teeth with different outcome parameters have shown that GE achieves comparable success rates in the hands of practitioners with different levels of experience, and that it has tooth substance preservation advantages over CONV [7].

In contrast to monochrome 3D-printed teeth in a lab setting, extracted teeth provide important additional orientation factors, such as the ability to distinguish secondary and tertiary dentin by color. Similar to the concept of roadmap recognition, access to the canal can be found by differentiation based on dentin layers, consistencies and anatomical landmarks [8].

In order not to deprive the CONV-operator of this advantage and to allow a more direct comparison with the clinical situation, the aim of this study was to compare the results of GE and CONV for the preparation of endodontic access cavities in extracted human teeth with PCC.

## Methods

### Tooth selection and model fabrication

**Fig. 1:**
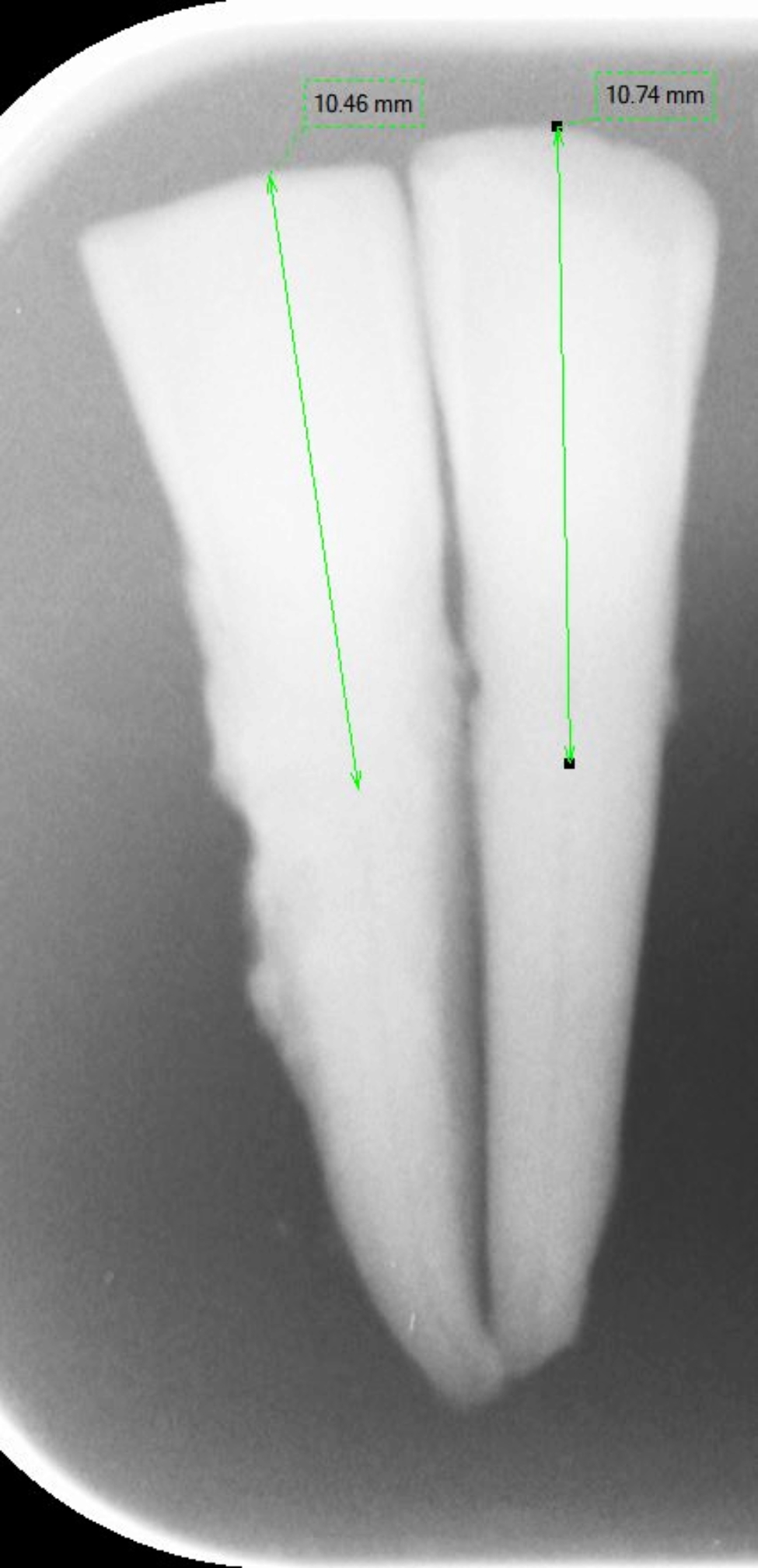
Two-dimensional X-ray of a matched pair of teeth. The incisal-pulpal distance of matched teeth could not differ by more than 10%

The use of human teeth extracted for reasons unrelated to this study was approved by the local ethics committee (protocol number EKNZ UBE-15/ 111) and is in line with the principles of the Declaration of Helsinki. The sample size was based on previously published studies [7, 9]. Overall, 108 human extracted calcified teeth (36 canines and 72 incisors, 36 maxillary and 72 mandibular teeth, which have been stored in 0.1% thymol solution and further processed without any association of patient data) were matched in pairs by two independent examiners based on the following criteria: tooth type, crown and root length, existence of fillings, and degree of calcification as determined based on two-dimensional (Digora Optime, Soredex, Milwaukee, Wisconsin, United States) and, later, 3D images (Accuitomo 170; Morita Manufacturing Corp, Kyoto, Japan). Particular care was taken to ensure that tooth length and pulp-incisal distance did not differ by more than 10% between pairs (Fig. 1).

Only teeth in which the pulp had retracted to at least 2 mm below the deepest part of the cementoenamel junction were included.

The tooth pairs were divided in two groups and supplemented with premolars and molars to produce physiological maxillary or mandibular arch models. In order to fit the models in a dental dummy (Dentsply Sirona, Charlotte, North Carolina, USA), a customized base was designed (PalaXpress, Kulzer, Hanau, Germany).

### Model / intraoral scanning and 3D imaging

Images of the models were captured using an intraoral scanner (TRIOS 3 Basic, 3Shape A/S, Copenhagen, Denmark) and saved in standard tessellation language (STL) format. Furthermore, a cone-beam computed tomography (CBCT) scan (Accuitomo 170; Morita Manufacturing Corp, Kyoto, Japan) of each model was obtained at 80KV and 6 mA using a voxel size of 250 μm and a field of view (FOV) of 10 × 10 cm. The CBCT images were exported in Digital Imaging and Communications in Medicine (DICOM) format for further planning.

### Access cavity preparation

In the CONV group, nine models were transferred to an independent endodontic specialist with 18 years of experience. To simulate a clinical setting, the specialist placed the models in a dental dummy and performed CONV on the anterior teeth (canine to canine; n = 54) using a high-speed contra-angle handpiece (1:5, KaVo Master Series; KaVo Dental GmbH, Biberach, Germany) with a standard cylindrical diamond bur with round edges and a diameter of 1.0 mm (837 KR; Intensiv SA, Montagnola, Switzerland) and an operating microscope for optical magnification (OPMI Proergo; Carl-Zeiss AG, Jena, Germany). Once a root canal orifice was located, accessibility was checked with an ISO 10 file (C-Pilot; VDW GmbH, Munich, Germany). Two- and three-dimensional X-ray images were available to the operator for diagnostic analysis purposes.

In the second group (GE), the other nine models were transferred to a general dentist with 4 years of professional experience but no previous experience with GE. The practitioner received a brief introduction to the GE technique in accordance with the clinical treatment protocol of Connert et al. [10]. Care was taken to leave the planning and procedure entirely to the discretion of the dentist.

**Fig. 2:**
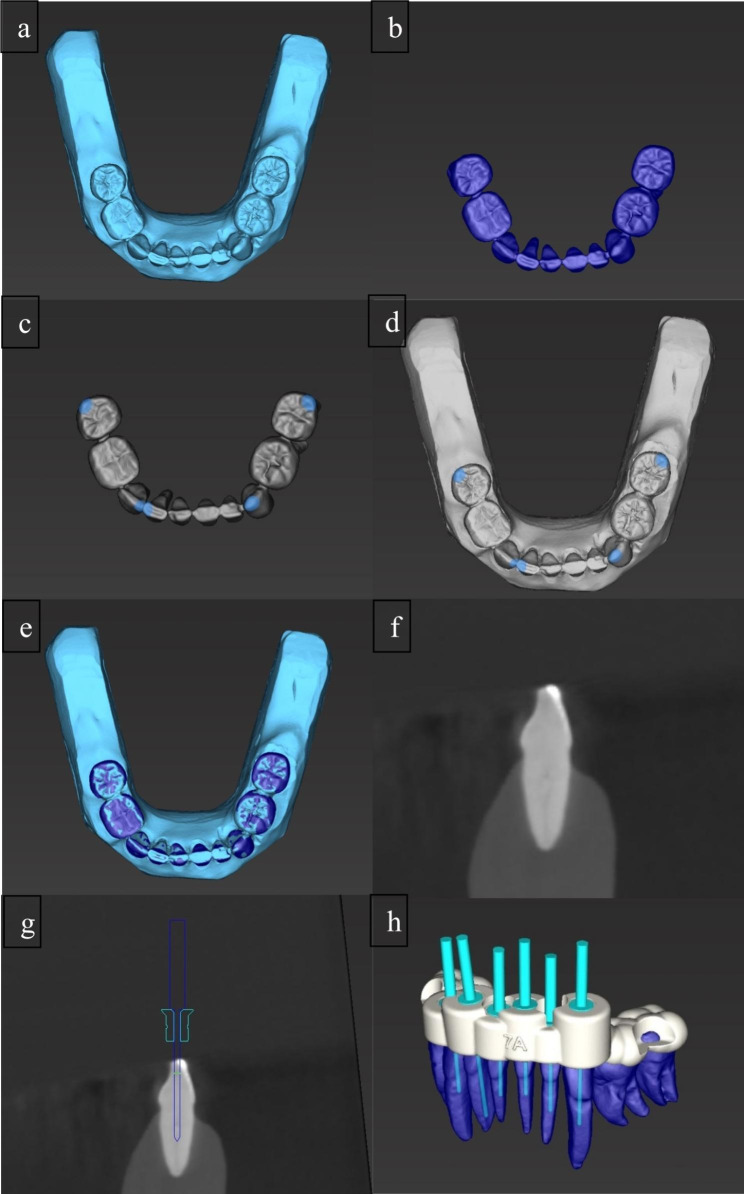
The intraoral scan (a) and the CBCT image (b) were matched in the planning software (c to e). Virtual drills were placed to the root canal orifices to provide a straight-line access (f and g) and a template was designed (h)

Templates were digitally designed using planning software (coDiagnostiX, Dental Wings Inc., Montreal, Canada). For this purpose, the operator matched the intraoral scan and CBCT data to create a combined digital model and then planned an access cavity by placing a virtual drill to the orifice of the calcified root canal (Fig. 2). A digital template was then created, exported in STL format and sent to an external dental laboratory, where it was fabricated from polymethyl methacrylate discs (98.5 mm; Yamahachi Dental, Gamagori, Japan) by computer-aided manufacturing. Sleeves were inserted into each fabricated template (StecoGuide Endo-Sleeve; Steco-System-Technik, Hamburg, Germany), providing the final guide for a carbide drill with a diameter of 1.0 mm (ATEC Endoseal, Steco-System-Technik). Similar to the CONV group, models were placed in a dental dummy to simulate clinical conditions. Before ACP, enamel had to be removed with the help of the templates. The entry point was marked and enamel was removed using a diamond bur (837 KR; Intensiv SA, Montagnola, Switzerland) in a contra-angle handpiece (EXPERTmatic, KaVo Dental GmbH). Subsequently, the access cavity was prepared with a contra-angle handpiece at 10,000 rpm. Throughout this process, a hand file was repeatedly used to check whether the root canal could be found ahead of reaching the final position of the drill. The operator was free to use the same optical magnification aids as the specialist. Furthermore, both operators were free to use additional intraoral radiographs during ACP for orientational purposes.

If the root canal could not be detected according to the planning, the axis of the drill had to be corrected. As in the first group, accessibility was verified with an ISO 10 file (C-Pilot, VDW). A new bur was used for each model.

For both operators, the time from the start of preparation until the root canal was found was measured.

### Substance loss measurement and statistical analysis

**Fig. 3:**
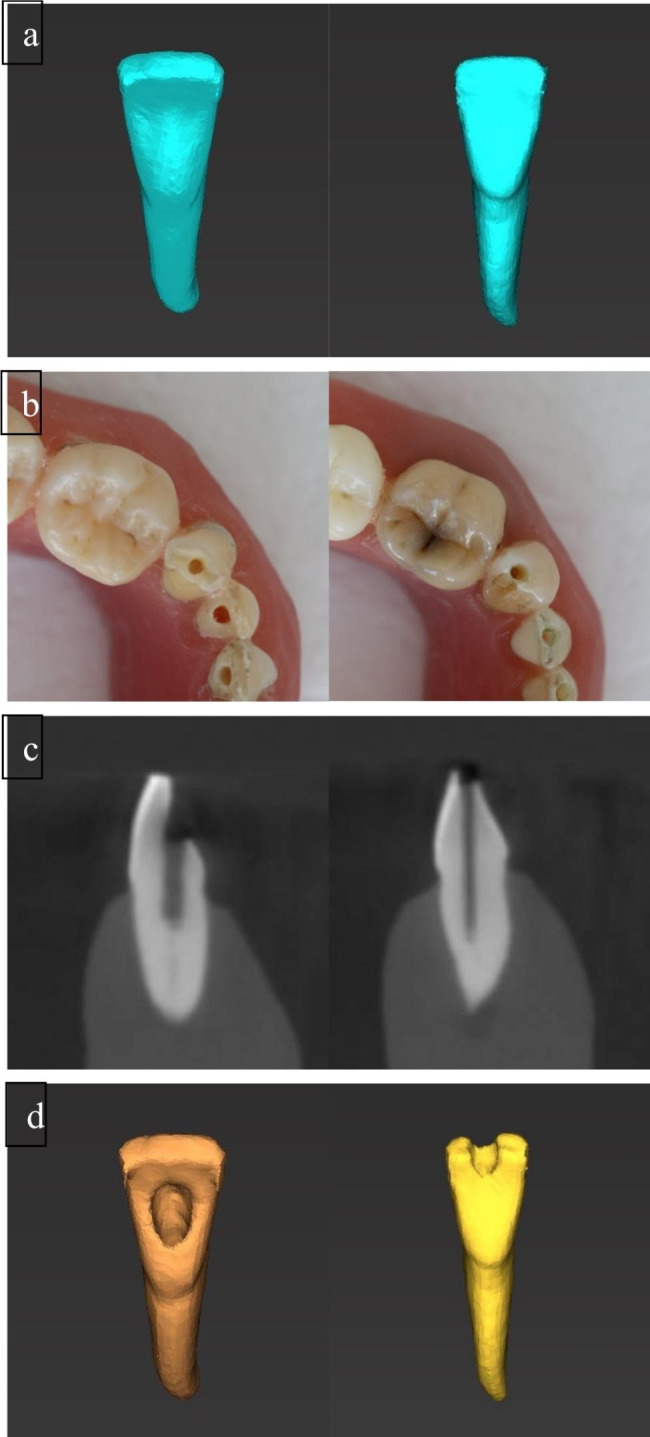
Preoperative single-tooth segmentation of a lateral incisor for volume determination (a), study model with performed ACP (b), postoperative CBCT scan of the access cavities (sagittal plane) (c) and postoperative single-tooth segmentation for volume determination in CONV (left) versus GE group (right) (d)

First, the volume of each tooth was calculated from the preoperative CBCT data by segmenting the tooth from the rest of the scan in a step automatized by the planning software. After ACP, each model was re-scanned by CBCT using the same settings as in the initial scan. Subsequently, tooth volumes were measured again (Fig. 3) and compared with pretreatment values to calculate hard substance loss resulting from ACP. Pre- and postoperative volume measurements were conducted by two investigators not involved in the experiment procedure. It was ensured that the grayscale thresholds were set to exactly the same values for the pre- and postoperative image data, resulting in corresponding automatic volume calculations by the software for both investigators. Microsoft Excel (Microsoft Corporation, Seattle, Washington, USA) was used to analyze the results, and the overlap of 95% confidence intervals (CIs) was used to test the statistical significance of differences between the groups.

## Results

Both GE and CONV detected all root canals in their respective group of 54 teeth. There was no case of root perforation in either group. Regarding mean procedural time, a slight advantage of CONV (205.9 s; CI: 163.3-248.5 s) by a specialist over GE (264 s; CI: 231.9-326.8 s) by a general dentist was observed, but the difference was not statistically significant.

The dentist performing GE required 31 additional X-rays for 14 teeth, whereas the specialist performing CONV managed without any additional radiographs.

There were no significant differences in mean substance loss between CONV (22.8 mm^3^; CI: 15.9–29.6 mm^3^) and GE (22.6 mm^3^; CI: 15.9–29.6 mm^3^ vs. 17.6–27.5 mm^3^, respectively). Table 1 summarizes the outcomes for root canal detection, substance loss, procedural time and additional radiographs by procedure and operator.

**Table 1:** Outcomes for root canal detection, number of additional periapical radiographs required, mean substance loss and procedural time for CONV versus GE by an endodontic specialist versus general dentist, respectively

Method (operator)	Detected canals (n)	Substance loss (95% CI) (mm)	Procedural time (95% CI) (s)	Additional periapical radiographs (n)
CONV (specialist)	54/54	22.8 (15.9–29.6)	205.9 (163.3–248.5)	0
GE (general dentist)	54/54	22.6 (17.6–27.5)	264 (231.9–326.8)	31

Figure 3 shows representative results for both techniques.

## Discussion

The results of this study show 100% success in locating the calcified root canals by both methods. These results are consistent with the high accuracy and success rates for GE reported in several previous studies [9–13]. Furthermore, our comparative analysis showed no significant difference in hard tissue loss between CONV by a specialist in endodontics and GE by a general dentist.

This is in contrast to previous studies comparing the two procedures for substance loss. Leontiev et al. and Connert et al. used 3D printed teeth made of a single material in their studies [7, 9]. Important guiding features, such as the natural and pathological coloration, variations in the consistency of the dentin and anatomical landmarks are then missing. For example, a calcified root canal can be identified as a gray translucent area surrounded by dark tertiary dentin [8]. Such additional information helps experienced endodontists locate root canals using CONV. The results of the present study indicate that a specialist was able to accurately locate root canal orifices by “road-mapping”.

Considering the aforementioned studies from Connert et al. and Leontiev et al., these findings support the assumption that knowledge of anatomical landmarks is key to successful endodontic treatment of teeth with PCC.

Nevertheless, conventional treatment of teeth with PCC involves considerable complications. Kvinnsland et al. estimated that the failure rate due to perforation is 20% in such cases [14]. Cveck et al. also reported a failure rate of 20% [15], although other studies indicate that trained specialists can achieve success rates of 89% [5].

Treatment durations did not differ significantly between the two groups (mean 4.4 min vs. mean 3.4 min), although the GE group showed a tendency towards longer times. GE involves a greater number of individual steps compared to CONV, such as the removal and reinsertion of the splint when checking if the root canal could be accessed with a hand-file. In addition, the longer procedure time for GE may be due to the increased effort required when the technique initially failed to locate the root canal at the anticipated drilling depth, requiring unplanned further adjustments. However, the time requirements for GE are comparable to those observed in similar studies [5, 9].

A previous study by Connert et al. with 3D printed teeth demonstrated a significantly better time performance for GE over CONV, in contradiction to the results of this study [9]. The authors suggest that extracted human teeth may provide more information for accurate and fast treatment.

The time required in the virtual preparation planning phase was not included in the GE procedural time calculations. Connert et al. reported an average of 9.4 min for virtual planning, which is acceptable considering that the total procedural time for CONV exceeds 30 min in 40% of cases and lasts up to 60 min [5, 9, 11].

There were distinct differences in the number of additional radiographs required. Whereas the specialist required none for CONV, the general dentist performing GE required n = 31 additional radiographs for 14 teeth. However, nine images only served to confirm the detection of the root canal. Under clinical circumstances, this would have been avoided by using an apex locator in combination with a measuring radiograph. Further images were taken if the root canal had not been located after reaching the planned drilling length. This was a precautionary measure to confirm that the drilling axis was maintained as planned. The other radiographs were taken to correct the drilling axis, to exclude the possibility of perforation (which could not be done in any other way due to the hard consistency of the PMMA base of the models), and to further confirm the drilling axis after already exceeding the planned drilling depth. Despite the low radiation exposure of dental radiographs (4.5 µSv [16]), the need for additional radiographs must be regarded as a disadvantage of GE in this study.

In recent years, GE has been established as a successful endodontic treatment alternative [1]. Although GE has received wide attention and dissemination in the field of endodontics, it currently is still regarded as a niche application that is mostly used by endodontic specialists.

Disadvantages include the limited ability to use GE in the posterior region and in teeth with thin roots, increased radiation exposure and additional costs associated with planning and fabrication of a template [17]. However, some of these problems have been solved by recent advances, such as thinner drills for precise preparation in thin roots. Moreover, the increased radiation exposure associated with CBCT is justified by higher therapeutic success rates [10], and recent modifications to the GE procedure have demonstrated increasing success in the treatment of root canals in the posterior region or in patients with reduced mouth opening [18].

GE has also become more relevant due to the increasing availability of CAD/CAM technologies which, in turn, has led to price reductions reflected in decreasing treatment costs for GE. Likewise, the use of 3D printers for GE results in lower cost with sufficiently high accuracy [19].

The technique of guided implantology from which GE was derived is widely recognized as an accurate and reliable method in the field of oral surgery [6]. With the help of guided implantology, even inexperienced practitioners can safely plan and treat complicated cases. Likewise, our results indicate that even in the hands of general dentists who are not endodontic specialists, GE may provide good treatment outcomes with superior treatment safety. Although observed treatment times for generalists are slightly higher than those for specialists, key advantages of GE are its predictability and ready availability in general practice. Based on the outcomes obtained in the present study, GE allows general dentists to produce endodontic treatment results in teeth with PCC at the level of a specialist.

This in vitro study has certain limitations, primarily due to the dependence of the results on the operator. Connert et al. demonstrated significant differences in the performance of users with different levels of experience and emphasized the superiority of specialists over inexperienced dentists using CONV. However, the same study did not find significant differences in performance between users in GE [9]. Therefore, this study focuses on the comparison between GE and specialists using CONV. The circumstance that GE was performed by a general dentist emphasizes the broad applicability of the technique.

A limitation of this study is the lower resolution of the CBCT scans used for volume measurement. This is due to a large FOV used to ensure precise matching between the intraoral scan and the CBCT scan itself in the planning software. However, the measurement accuracy is lower, but for the comparison of the two investigated methods, the absolute value is of minor importance. Since this is an *ex-vivo* study and, in contrast to a clinical situation, there are no motion artifacts, good imaging quality was ensured despite the high voxel size. In a clinical setting, a smaller field of view as well as the resulting reduced radiation exposure would be preferable.

To our knowledge, this is the first study comparing GE and CONV in human teeth with severe PCC. Most ex vivo studies in the literature have focused on the proof of concept and accuracy of GE in small samples and/or in teeth without PCC [11, 12, 20]. However, these results cannot be transferred to the accuracy in a clinical setting, but it does allow comparison of different methods while reducing bias as much as possible. Therefore, further clinical studies are needed to confirm these results.

## Conclusions

GE and CONV achieved comparable success rates for root canal location and ACP and comparable results for procedure time and tooth structure loss in canines and incisors with PCC. Although additional radiographs were often needed for GE, both methods achieved similar success rates despite the different levels of experience of the users.

### Electronic supplementary material

Below is the link to the electronic supplementary material.

Supplementary Material 1

## Data Availability

All data generated or analyzed during this study are included in supplementary file 1.

## List of abbreviations

ACP: Access cavity preparation
CBCT: cone-beam computed tomography
CI: Confidence interval
CONV: Conventional access cavity preparation
DICOM: Digital imaging and communications in medicine
FOV: Field of view
GE: Guided endodontics
PCC: Pulp canal calcification
STL: Standard tessellation language
